# Tubal Stump Ectopic Pregnancy After IVF-ET in Patients Who Underwent Salpingectomy or Adnexectomy: A Qualitative Systematic Review

**DOI:** 10.3390/medicina62010083

**Published:** 2025-12-31

**Authors:** Massimo Criscione, Giorgio Maria Baldini, Elisa Sanna, Laura Saderi, Giovanni Sotgiu, Mario Palumbo, Marco Petrillo, Giampiero Capobianco

**Affiliations:** 1 Gynecologic and Obstetric Clinic, Department of Medicine, Surgery and Pharmacy, University of Sassari, 07100 Sassari, Italy; massimo.crisc@gmail.com (M.C.); elisa-sanna@hotmail.it (E.S.); marco.petrillo@gmail.com (M.P.); capobia@uniss.it (G.C.); 2 Obstetrics and Gynaecology Unit, Department of Interdisciplinary Medicine (DIM), University of Bari “Aldo Moro”, 70121 Bari, Italy; 3 Clinical Epidemiology and Medical Statistics Unit, Department of Medicine, Surgery and Pharmacy, Sassari University, 07100 Sassari, Italygsotgiu@uniss.it (G.S.); 4 Department of Public Health, School of Medicine, University of Naples “Federico II”, 80131 Naples, Italy; mario.palumbo@unina.it

**Keywords:** ectopic pregnancy, salpingectomy, tubal stump pregnancy, IVF-ET, heterotopic pregnancy, cornual pregnancy

## Abstract

*Background and Objectives:* Ectopic pregnancy (EP) is a life-threatening medical and surgical condition. Tubal stump EPs and heterotopic pregnancies can occur after in vitro fertilization-embryo transfer (IVF-ET), even after salpingectomy. The purpose of this study is to investigate the risk factors, diagnosis, and treatment of tubal stump EPs after IVF-ET in patients with prior salpingectomy or adnexectomy. We also aim to evaluate the intrauterine pregnancy (IUP) outcome in cases of heterotopic pregnancy in this population. *Materials and Methods:* This systematic review (PROSPERO CRD42023352959) followed PRISMA guidelines. A literature search of MEDLINE^®^, Scopus, Web of Science, and clinicaltrials.gov was conducted on 30 April 2024. We included studies on tubal stump EP after IVF-ET in patients with previous salpingectomy or adnexectomy and created a qualitative summary. *Results:* We included 40 studies reporting on 57 patients (58 EP episodes). Most patients (69.0%) had prior bilateral salpingectomy. Tubal rupture occurred in 69.6% of cases, with 69.0% of these cases reporting hemoperitoneum. Abdominal pain was the most frequent symptom (71.7%). Heterotopic pregnancy occurred in 60.0% of cases (82.7% singletons). The IUP outcome was delivery in 81.9% of cases, with 95.5% of singletons delivering at term, compared with 40.0% of twins. The surgical approach (laparoscopy vs. laparotomy) did not change the IUP outcome. Tubal stump excision (74.1%) was the most common treatment. Overall, the certainty of the evidence was judged as moderate to very low according to the GRADE-CERQual approach, mainly due to small sample sizes, observational designs, and heterogeneity among studies. *Conclusions:* This review, the first on this topic, provides key data for counselling patients with a tubal stump heterotopic pregnancy. Despite its rarity, close follow-up until 8–10 weeks is recommended for IVF-ET patients with positive β-hCG, monitoring for abdominal pain. Successful management (expectant, medical, or surgical) should be guided by β-hCG levels and ultrasound findings (e.g., absence of heartbeat). Medical treatment shows encouraging obstetric outcomes and warrants further research.

## 1. Introduction

Ectopic pregnancy (EP) is defined as the implantation of the blastocyst outside the endometrium of the uterine cavity, representing about 2% of all pregnancies [1]. The most common localization is the fallopian tube, mainly the ampullary (70%), isthmic (12%), and fimbrial (11%) portions, with rarer occurrences in the interstitial tract, ovary, abdomen, or cervix [2]. Without early diagnosis, EP can result in tubal rupture, hemorrhage, and death, accounting for 75% of first trimester maternal deaths [3,4]. A tubal stump pregnancy is a rare form of EP that occurs within the remnant of the fallopian tube after salpingectomy [5], and its incidence has been reported between 0.4% and 1.16% [6,7].

Although frequently misclassified as “cornual” pregnancy, this term should strictly refer to implantation in a uterine malformation, such as a rudimentary horn or bicornuate uterus [8]. The increasing use of assisted reproductive technologies (ARTs) has modified the epidemiology of EP: up to 7.8% of pregnancies after in vitro fertilization and embryo transfer (IVF-ET) are ectopic [9], and heterotopic pregnancies—coexisting intrauterine and extrauterine gestations—occur in 1–2.9% of IVF-ET cycles [10,11,12]. The largest ART database from the USA reported an incidence of heterotopic pregnancy of only 0.09% among 553,577 IVF pregnancies, confirming its rarity after bilateral salpingectomy [13]. Multiple mechanisms contribute to EP after IVF-ET. These include maternal factors, such as tubal disease, prior surgery, endometriosis, pelvic inflammatory disease (PID), or smoking; and technical factors, such as high transfer medium volume, multiple embryo transfer, or transfer performed too close to the uterine horn [14,15,16,17,18]. Despite bilateral salpingectomy or cornual occlusion, interstitial pregnancies have been reported even in women without visible tubes [18]. Previous ectopic pregnancy remains the strongest predictor for recurrence, increasing from 10% after one EP to over 25% after two or more [19].

Risk factors for EP can be classified as high (previous EP, tubal sterilization, intrauterine device [IUD] use, PID, salpingitis isthmica nodosa), moderate (chlamydia infection, infertility, ART, multiple partners, smoking), or low (gamete intrafallopian transfer [GIFT], age < 18 or >35 years, vaginal douching) [20,21]. Surgical procedures such as salpingostomy, fimbrioplasty, or tubal reanastomosis also increase EP risk [22]. Conversely, bilateral salpingectomy before embryo transfer is often performed to improve IVF outcomes in patients with hydrosalpinx or tubal pathology [23]. Given these observations, tubal stump EP following IVF-ET represents an exceptional yet clinically significant event, frequently associated with heterotopic pregnancy. To the best of our knowledge, no previous systematic review has specifically analyzed its etiology, risk factors, and the prognosis of the concomitant intrauterine pregnancy. The present study aims to evaluate tubal stump ectopic pregnancy after IVF-ET in women with prior salpingectomy or adnexectomy, assessing diagnostic features, management strategies, and outcomes for both the ectopic and concomitant intrauterine gestations.

## 2. Materials and Methods

### 2.1. Study Registration and Protocol

This systematic review was conducted in accordance with the Preferred Reporting Items for Systematic Reviews and Meta-Analysis (PRISMA) Guidelines 2020 [24]. The PRISMA checklist is provided in the Supplementary Files (Table S1). Using the PICO (population/patients, intervention/exposure, control/comparison, and outcome) strategy, the studies that meet the following criteria were included in the study. The PICO strategy is presented in Supplementary Table S2. The review protocol was defined a priori and registered in the International Prospective Register of Systematic Reviews (PROSPERO; CRD42023352959). The protocol has not been published as a separate manuscript. No amendments to information were produced at registration or in the protocol.

### 2.2. Information Sources and Search Strategy

A literature search of MEDLINE^®^, Scopus, Web of Science, and clinicaltrials.gov was conducted on 30 April 2024. The electronic search strategy was jointly developed by a gynecologic oncologist with clinical expertise in ectopic pregnancy and an epidemiologist experienced in systematic reviews. The search strategy was not subjected to a formal PRESS (Peer Review of Electronic Search Strategies) assessment. We systematically searched the three electronic databases using a search strategy available in the Supplementary Materials; the Mesh terms used were: “Salpingectomy” [Mesh] OR “Fallopian Tubes/surgery” [Mesh] OR salpingectomy OR salpingectomies OR adnexectomy OR adnexectomies OR tubectomy OR tubal stump OR “tubal remnant” OR “tubal segment” OR “tubal surgery” OR “post-salpingectomy” OR stump) AND (“Pregnancy, Ectopic” [Mesh] OR “Pregnancy. Tubal” [Mesh] OR “Pregnancy” [Mesh] OR pregnancy OR pregnancies OR gestation OR gestational OR “heterotopic pregnancy” OR “tubal stump pregnancy” OR “ectopic pregnancy” OR “Fertilization in Vitro” [Mesh] OR “Embryo Transfer” [Mesh] OR “In Vitro Fertilization” OR IVF OR “IVF-ET” OR “assisted reproduction” OR “assisted reproductive technology”. There were no language or publication date restrictions for the included studies. Literature written in languages other than English has been translated.

### 2.3. Eligibility Criteria and Study Selection

**Figure 1:**
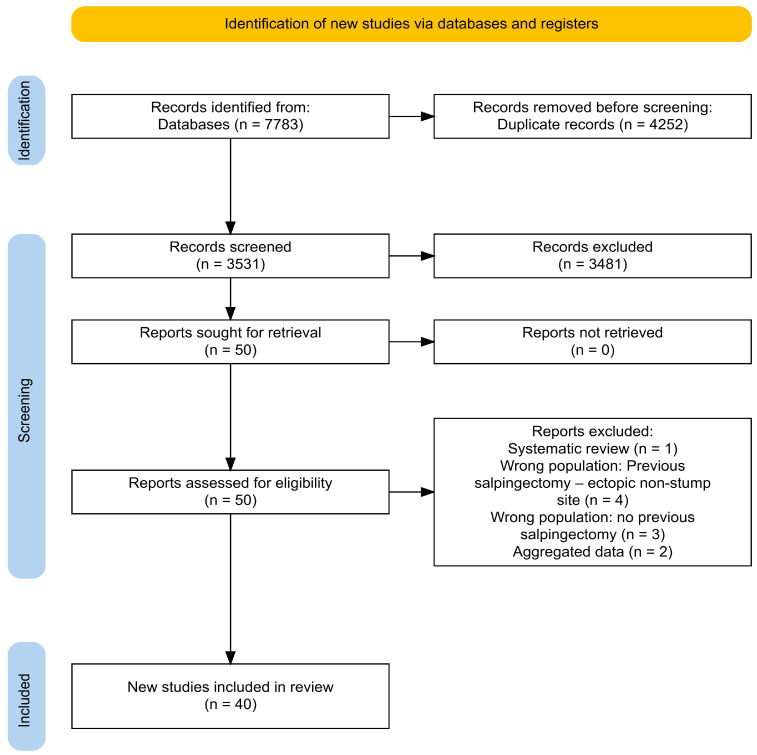
Adapted Preferred Reporting Items for Systematic Reviews and Meta-Analysis (PRISMA) Guidelines 2020.

The eligibility criteria for inclusion in the present review were defined a priori to ensure consistency and methodological rigor. We included prospective and retrospective studies, as well as case reports, case series, letters to the editor, and video articles that provided relevant clinical information. The study population of interest comprised patients diagnosed with ectopic pregnancy located in the tubal stump following in vitro fertilization and embryo transfer (IVF-ET) in women who had previously undergone mono- or bilateral salpingectomy or monolateral adnexectomy. Studies were excluded if they were review articles, systematic reviews, discussion papers, non-research editorials, qualitative studies, conference abstracts, or animal studies. Full-text articles were excluded for the following reasons: wrong population (e.g., no previous salpingectomy or ectopic pregnancy in a non-stump site), aggregated data preventing patient-level extraction, or study type (systematic reviews). A detailed list of excluded studies with specific reasons is provided in Supplementary Table S3 [25,26,27,28,29,30,31,32,33,34]. To identify eligible studies, two reviewers (M.C. and E.S.) independently screened titles and abstracts and then reviewed the full texts of the studies that were considered to potentially meet the inclusion criteria. The selection process flow diagram is available in Figure 1 (PRISMA flow diagram using an online app for producing PRISMA 2020-compliant flow diagrams available at https://estech.shinyapps.io/prisma_flowdiagram/ (accessed on day 11 November 2025)) [35] Discrepancies were resolved by discussion and consensus, involving a third reviewer (G.S.) when necessary. In case of missing information, authors were contacted.

### 2.4. Data Collection and Quality Assessment

For data extraction, two authors independently reviewed all eligible articles and collected individual patient-level data using a standardized spreadsheet (see Table S4 for index data and collecting rules). The extraction template was initially piloted by two users on a set of five randomly selected cases and adjusted as necessary. We attempted to collect technical parameters of the embryo transfer (e.g., catheter placement depth, embryo stage), but these were sporadically reported and could not be included in the final analysis. Two reviewers (M.C. and E.S.) independently assessed the quality of the studies. Risk of bias was assessed using an ad hoc checklist derived from the 2017 Joanna Briggs Institute (JBI) critical appraisal tools for case reports and case series [36], in which overlapping items were merged, non-applicable items removed, and selected questions rephrased to reflect key sources of bias for this clinical condition. The bias analysis is represented graphically in the Supplementary Materials (Figure S1).

### 2.5. Data Analysis and Synthesis

Primary outcomes are the evaluation of: treatment and intrauterine pregnancy outcome (laparoscopy, laparotomy, medical treatment), gestational age at diagnosis (days) in ruptured vs. unruptured pregnancy, intrauterine outcome in heterotopic pregnancies, and delivery at term in ruptured vs. unruptured pregnancy. Secondary outcomes include: length of stay stratified by surgical procedure (laparoscopy vs. laparotomy), symptoms, and median stump larger diameter in ruptured vs. unruptured pregnancy.

Given the marked clinical and methodological heterogeneity across the included reports—in terms of study design (case reports, small case series, observational studies), settings, and reporting formats—we did not attempt any formal quantitative meta-analysis. Qualitative data were summarized with absolute and relative (percentages) frequencies, whereas quantitative ones were summarized with means (standard deviations, SDs) or medians (interquartile ranges, IQRs), depending on their parametric distribution. Patients’ clinical findings according to pregnancy characteristics were compared using the chi-square test for categorical variables and the Mann–Whitney U test for quantitative variables. A *p*-value of <0.05 was considered to indicate a statistically significant difference. Statistical analyses were performed using STATA version 17 (StataCorp LLC, College Station, TX, USA) and were used in an exploratory manner only.

We assessed the confidence of the qualitative findings of the systematic review through the GRADE-CERQual Interactive Summary of Qualitative Findings (iSoQ). Confidence ratings refer to how likely it is that a qualitative statement (e.g., “most reported cases presented with…”) adequately reflects the body of evidence rather than comparative treatment effects or generalizability to all clinical settings. In line with GRADE-CERQual guidance, we assessed confidence in each descriptive review finding across the four standard domains (methodological limitations, coherence, adequacy, and relevance). Given the extreme rarity of tubal stump ectopic pregnancy after IVF-ET, we did not downgrade confidence solely because the primary data derive from observational case reports and small series; instead, we considered whether, for each specific finding, methodological limitations were minor, data were rich and directly relevant, and patterns were highly consistent across studies. The Summary of Qualitative Findings table (iSoQ table, evidence profile, and GRADE-CERQual worksheets) is fully public and available on the iSoQ database and in the repository materials [37].

## 3. Results

**Figure 2:**
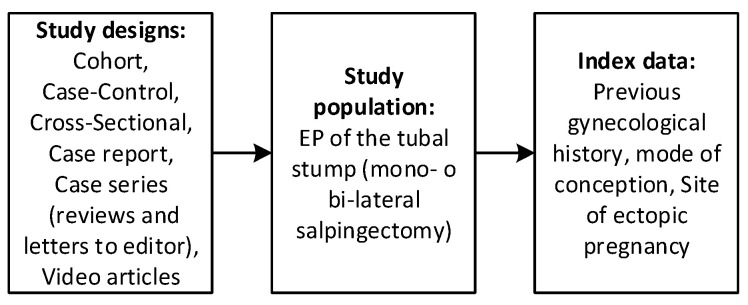
Selection process—Study designs, study population, and index data eligible for this review.

The literature search found 7783 records (SCOPUS (n = 1779); Medline^®^ (n = 3966), WEB OF SCIENCE (n = 2038)). After removing 4252 duplicates, 3531 articles were reviewed based on their title and abstract. Among them, 51 full texts were assessed for inclusion, while 51 articles were excluded based on their full text. Finally, 40 studies that met the research criteria were identified and included in the analysis. The study screening and selection process is summarized in Figure 2. No clinical trials were found on the clinicaltrials.gov (NIH) register.

The 40 included studies report discrete data for 57 patients (58 cases of ectopic pregnancy of tubal stump).

The literature lacks good evidence regarding ectopic pregnancies on tubal stump after IFV-ET, also due to the impossibility of carrying out randomized clinical trials. This study is the only study that provides the patient, in a counseling setting, with information regarding the outcome of intrauterine pregnancy in case of heterotopic pregnancy in case of extrauterine pregnancy on tubal stump following IVF-ET.

The 40 included studies report discrete data for 57 patients (58 cases of ectopic pregnancy of tubal stump). Two articles report the same cases: the study by “Di Tucci et al.” was excluded because it was published after “Piccioni et al.” [38,39]. An email was sent to the authors, but no response was received. The authors of two studies providing aggregated data were contacted to receive discrete patient data, but we received no response [26,40]. The PRISMA flow-chart is presented in Figure 1 [41].

### 3.1. Study Characteristics

The 40 included studies provided discrete data on 57 patients (58 cases of tubal stump ectopic pregnancy). The main features of the included studies are presented in summary in Table 1 (bias analysis of the included studies is available Supplementary Material, Table S5 [7,14,15,42,43,44,45,46,47,48,49,50,51,52,53,54,55,56,57,58,59,60,61,62,63,64,65,66].

### 3.2. Population Characteristics

Ruptured pregnancy occurred in 39/56 cases (69.6%), while 17/56 (30.4%) were unruptured. From the analysis of the data obtained, it was found that the mean (±SD) age of the patients under study was 32.6 (±3.7) years. These were mostly patients who had already had at least another pregnancy (90.0% of the patients had gravidity ≥ 1), but almost 76.5% of the patients had parity = 0. Furthermore, 73.4% of patients had previously had 1 to 5 ectopic pregnancies. The cause of previous salpingectomy, in the site of occurrence of tubal stump ectopic pregnancy, is in most cases previous tubal pregnancy (61.4%), while the second most common cause is hydrosalpinx (37.5%). Only two patients had previous PID. Most patients (69.0%) had undergone bilateral salpingectomy. As for bilateral salpingectomy, no significant difference in EP side was reported between right (18/40 (45.0%)) and left (22/40 (55.0%)). Only one patient presented bilateral ectopic pregnancy. Regarding the embryo transfer technique, the median of transferred embryo was 2 (IQR 2–3), fresh embryo 25.0%, frozen 20.0%, and not specified in 52.5% of cases. Population characteristics, ultrasound characteristics, heterotopic pregnancy outcomes, and treatment are available in Table 2. Characteristics of IVF-ET and EP are available in Table 3.

### 3.3. Diagnosis, US Outcomes, Symptoms

Median (IQR) gestational age at diagnosis was 45.5 (35–56) days. In 72.0% of cases there was ultrasound (US) evidence of fetal heartbeat, which was present in 91.7% of cases of pregnancy without rupture and in 53.9% of ruptured pregnancies (*p*-value 0.07). Median US CRL was 8.8 mm (IQR: 6–14), median of the gestational sac was 14 mm (IQR: 11–17), and median stump width was 25 mm (IQR: 20–33). An interesting finding concerns the diameter of the tubal stump, although without a statistically significant relationship, in ectopic pregnancies with rupture and those without rupture: median (IQR) stump larger diameter was 16 mm (15–17) for unruptured pregnancy versus 25 mm (21–55) in ruptured pregnancy (*p*-value 0.07). The median interval between salpingectomy and occurrence of ipsilateral (or bilateral) EP varies between 36 months (IQR: 15.0–84.0 months) after unilateral salpingectomy and 12 months (IQR: 12–24.0 months) after bilateral salpingectomy, with a *p*-value of 0.17. When present, bleeding presented a median volume of 650 mL (IQR: 200–1350). The median perioperative hemoglobin was 8.5 g/dL. As noted, 30.4% of ectopic pregnancies occurred without rupture, while almost 69.6% presented rupture. Hypovolemic shock symptoms were significantly related to ruptured pregnancy (*p* = 0.01). Ectopic pregnancy rupture does not influence the obstetric outcome of intrauterine pregnancy; there is no increase in miscarriage (*p* = 1.00), nor does it change the gestational age at delivery (*p* = 0.58). Characteristics of ectopic pregnancy and ruptured pregnancy are available in Table 4.

Abdominal pain was reported by 71.7% of patients, making it the most frequent symptom. The others, in order of frequency, were vaginal bleeding (31.4%) and hypovolemic shock symptoms (23.5%). Only 4 patients out of 51 had the complete triad (amenorrhea, abdominal pain, and vaginal bleeding). Vaginal bleeding can be misleading as it can misdirect the differential diagnosis to miscarriage. Not enough data regarding β-HCG for heterotopic pregnancies (singleton and twin) was available to perform quantitative analysis. IU pregnancy outcome and characteristics of ectopic pregnancy (gestational age at diagnosis, symptoms, laboratory, ultrasound findings) are summarized in Table 5, Table 6 and Table 7.

### 3.4. Heterotopic Pregnancy (HP) Outcomes

Heterotopic pregnancy occurs in 60.0% of cases; of these, 82.7% are singletons and 15.2% are twins. In 81.9% of cases, the outcome of intrauterine pregnancy was delivery (cesarean section in 66.7% of cases, 6.1% vaginal delivery, and 9.1% unspecified type of delivery), while 18.2% of intrauterine pregnancies evolved into miscarriage. The caesarean section was elective in 54.6%, while in 31.8% it was performed as an emergency (in 13.6%, the mode of cesarean section is not specified). Twenty-five patients out of twenty-seven delivered healthy babies; in the remaining two cases it was not specified. Median birth weight is 2365 g (IQR: 1915–2814). Singleton birth median weight: 2365 g (IQR 1915–2814); twin birth median weight: 2490 g (IQR 1590–2500). Delivery at term occurred in 85.2% of all intrauterine pregnancies, of which singleton intrauterine pregnancies delivered at term in 95.5% of cases, compared with 40.0% of cases of twin intrauterine pregnancies. There are few data to correlate treatment with intrauterine pregnancy outcome. Three cases of medical treatment out of five and one case of expectant management were successful with full-term delivery of the intrauterine pregnancy. There is no difference between laparoscopy and laparotomy (undertaken in 51.7% and 41.4% of cases, respectively) with respect to the surgical treatment used. Heterotopic pregnancy characteristics and pregnancy outcomes are summarized in Table 7.

### 3.5. Treatment

Nearly 46.7% of patients underwent laparotomy after conversion from laparoscopy. Most of the surgical management consisted of tubal stump excision (74.1%), followed by cornuostomy (24.1%). Only in one case was hysterectomy necessary. There were no differences in the management of ectopic pregnancy with rupture and without rupture (Table 4). Two cases were treated surgically after failure of medical treatment: A single dose i.m. MTX regimen (50 mg/m^2^) evolved into operative laparoscopy [38] due to stump bleeding; after a sonographically guided intracardiac potassium chloride injection, a laparotomy was performed with a cornuostomy because of ectopic pregnancy bleeding. In this last case, the intrauterine pregnancy reached full term (37 weeks) and was delivered by a cesarean section [50]. Sentilhes, in case of failure to rupture and absence of heartbeat, proposes expectant management, leading to the resolution of the ectopic pregnancy and reaching full-term delivery at the 39th week with cesarean section, after uterine rupture due to labor [71].

### 3.6. Other Outcomes

Discharge occurred on average on postoperative day 4. Length of postoperative hospitalization (days): laparoscopy 1.5 (1–3) versus laparotomy 6 (4.5–7.0) (*p*-value: 0.06) (Table 8).

In addition to the obstetric short-term outcomes (e.g., outcome of intrauterine pregnancy), obstetric long-term outcomes should also be considered. In Maruthini, for the patient, after 1 year from elective uterine reconstruction, a fresh cycle of IVF (single embryo) was performed. The pregnancy test was positive, and a scan confirmed a viable intrauterine pregnancy at 7 weeks gestation. She remained well and was delivered by an elective caesarean section at term [64]. The findings certainty assessment via the GRADE CER-Qual assessment are available in Table 9.

## 4. Discussion

As ectopic pregnancy is among the foremost causes of maternal mortality during the first trimester of pregnancy, patients who undergo salpingectomy and IVF-ET should undergo a thorough transvaginal ultrasound with color Doppler mapping from the outset of pregnancy. This should combine imaging with serial β-hCG dosage, measuring and observing the size of the stump, CRL, the presence or absence of the fetal heartbeat, and other signs such as fluid in the Douglas pouch or symptoms indicating hemoperitoneum to guide conservative or surgical treatment. Obstetricians should be aware of the possibility of recurrent ipsilateral ectopic pregnancies so that careful measures can be taken to avoid fatal complications.

We do not have enough data to state that total salpingectomy can protect against the occurrence of tubal stump ectopic pregnancy compared with partial salpingectomy, nor are there similar data in the literature. Successful conservative management of interstitial pregnancies has been sporadically reported using intracardiac potassium chloride injection into the ectopic pregnancy [78,79]. Even though the entirety of the cases of heterotopic pregnancy treated with medical interventions in our study resulted in the successful delivery of the intrauterine pregnancy, the available data are insufficient to ascertain the efficacy of medical treatment with statistical significance.

Even hypothesizing mechanisms shared with the pathogenesis of scar pregnancy, it remains challenging to propose a minimum timeframe for proceeding with IVF-ET after salpingectomy. However, it is noteworthy that most extrauterine pregnancies following tubal stump occurred within a median timeframe of 12 to 36 months, depending on whether the salpingectomy was bilateral or unilateral, respectively.

In the event of a positive β-hCG dosage, it is recommended that all patients undergoing IVF-ET after salpingectomy undergo a routine ultrasound scan at the sixth week of pregnancy. Although two authors report a vaginal delivery [7,64], we recommend systematic cesarean section before labor to avoid a uterine rupture, as in Sentilhes [71].

A novel classification of ectopic pregnancies, which considers the surgical intervention involved in the management of the fallopian tubes, should be considered. Indeed, it appears judicious to establish a conceptual and terminological distinction between an ectopic pregnancy of the remnant resulting from partial salpingectomy (e.g., subsequent to tubal sterilization)—which is to be defined “proximal remnant ectopic pregnancy”—and the ectopic pregnancy of the isthmic portion resulting from total salpingectomy—instead defined “tubal stump”. Consequently, the term “tubal stump” should be reserved exclusively for ectopic pregnancies arising from total salpingectomy, even in cases involving cornual resection. Ectopic pregnancies of the distal tubal remnant following embryo transfer are not reported in the literature. This may be due to a different etiopathogenetic mechanism or to a bias linked to the population (women who choose tubal sterilization do not undergo ART).

### 4.1. Interpretation of Findings in Light of Risk of Bias

The interpretation of our results must take into account the quality of the included studies. Our risk of bias assessment, conducted using a modified JBI checklist, highlighted recurrent issues such as incomplete reporting of diagnostic work-up, variable follow-up duration, and lack of standardized definitions for “tubal stump” versus “cornual” pregnancy. Furthermore, the reliance on case reports and small case series introduces a significant selection bias, as cases with atypical presentations or severe complications are more likely to be published. Therefore, while our findings regarding the high rate of rupture and the favorable prognosis of the intrauterine pregnancy are consistent across reports, they should be interpreted with caution and be considered primarily representative of the published literature rather than the general population.

### 4.2. Risk Factors

It was originally thought that the risk of an ectopic pregnancy could be eliminated by removing the fallopian tubes [80]. However, even when the whole visible length of the tube is excised, the interstitial part will still remain within the uterine wall [81]. Cornual suture at the time of salpingectomy helps reduce the risk of interstitial pregnancy after ET [82]. Peterson et al. correlates the type of coagulation with increased risk of EP: bipolar coagulation RR 10 and unipolar coagulation RR 1.2 compared with a reference relative risk of 1 referring to postpartum partial salpingectomy [22].

Previous ectopic pregnancy is an anamnestic factor strongly associated with the recurrence of ectopic pregnancy; our study confirms that evidence since 73.4% of patients had previously had from 1 to 5 previous ectopic pregnancies. Particularly in our cohort, the main cause of previous salpingectomy, in the same side of occurrence of a tubal stump ectopic pregnancy, is in most cases previous tubal pregnancy (61.4%), followed by hydrosalpinx (37.5%). We report only two patients with previous PID. It seems important to limit the number of embryos transferred [83,84,85], in particular with patients who present risk factors for heterotopic pregnancy. In our study the median number of embryos transferred is 2, in line with recommendations [85].

### 4.3. Clinical Manifestations and Diagnosis

Difficulty in diagnosing these rare ectopic pregnancies may contribute to their high rate of complications. In 69.6% of cases there was evidence of ectopic pregnancy rupture with hemoperitoneum confirmed intraoperatively, with a median of 650 mL of blood loss (IQR 200–1350). The complete triad (amenorrhea, pelvic pain, vaginal bleeding) are aspecific because they are similar to those encountered in other clinical conditions and still not always present: in Ranji, the classic triad was present in only 27.7% of patients [86]. In Louis-Sylvestre, 54% of cases the heterotopic pregnancy was asymptomatic [87]. Among our results, only four patients had the complete triad (amenorrhea, abdominal pain, and vaginal bleeding). Abdominal pain was the most common symptom, referred by 38 out of 53 patients (71.7%), followed by vaginal bleeding (31.4%), while symptoms of hypovolemic shock were found in 12 out of 51 patients (23.5%).

#### 4.3.1. β-HCG and TVUS

A serum β-hCG level higher than 1500 mUI/mL is suggestive of EP in concomitant symptomatology after excluding intrauterine pregnancy (IUP). A single positive test of serum β-hCG level is of less clinical utility than serial tests. However, serial β-hCG level estimation may not be feasible in an emergency setting. A doubling of 50% in a 48 Hrs interval is noted in normal IUP, while it is less than 50% in failing IUP or EP. A doubling like that of IUP may be seen in up to 21% of EP.

**Figure 3:**
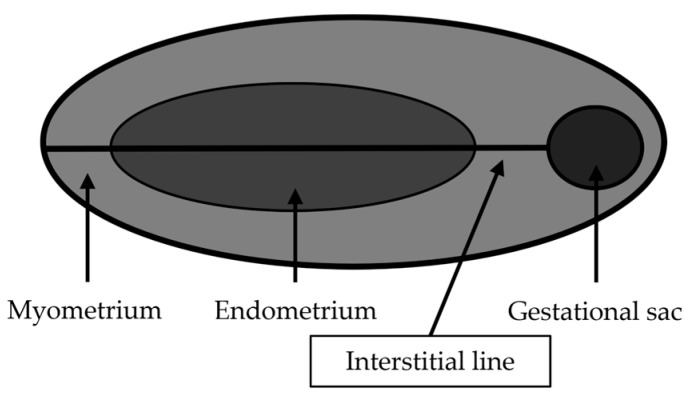
Interstitial line (modified from Bolaji, 2012 [93]).

TVUS is the initial and preferred way to diagnose HP, playing a crucial role in combination with other diagnostic criteria, in consideration of the nonspecific clinical presentation. In Wu et al. [88] a careful transvaginal ultrasound evaluation is suggested in symptomatic patients undergoing IVF-ET at any time. In asymptomatic patients, the first transvaginal ultrasound evaluation is suggested between the fourth and fifth week after ET (or the sixth and seventh week of gestation). TVUS has a reported sensitivity of 69–99% and specificity of 84–99.9% for EP diagnosis. Lack of intrauterine gestational sac, a thick echogenic cystic structure in the adnexa with a ‘‘ring of fire’’ appearance on Doppler, associated with the echogenic fluid in the pouch of Douglas are all imaging findings that may be seen on TVUS. A yolk sac or embryo with cardiac activity within the sac is highly specific [89,90]. The diagnosis of interstitial or tubal stump pregnancy is challenging, and this condition can be misdiagnosed as a normal IUP on ultrasound. The diagnosis of interstitial or tubal stump EP should be suspected when a gestational sac located in the lateral aspect of the uterine fundus is surrounded by less than 5 mm of myometrium at the lateral aspect of the uterus [91]. There are three ultrasound criteria to diagnose an interstitial pregnancy: eccentric gestational sac; thinning of the surrounding superficial myometrium; and interstitial line sign (visualization of the echogenic line that runs from the endometrial cavity to the corneal region, abutting the interstitial mass or gestational sac) (Figure 3). Ackerman believes that the interstitial line had better sensitivity (80%) and specificity (98%) than eccentric gestational sac location (sensitivity, 40%; specificity 88%) and myometrial thinning (sensitivity 40%; specificity 93%) [92,93].

The 3D scans are very useful in obtaining the coronal scans of the fundal region of the uterus, giving a better overview of the cornual regions of the uterus. The eccentric location and superior and lateral myometrial stripes are better and more easily visualized on coronal scans generated through 3D TVS, an infrequent achievement with 2D scans [94]. In our study 53.9% of patients with a ruptured ectopic pregnancy did not have a fetal heartbeat, while 91.7% of patients without a ruptured pregnancy had a heartbeat (*p*-value of 0.07). Early diagnosis of tubal pregnancy can be performed with ultrasonography and the measurement of the serum β-hCG level. Ultrasonographic findings suggestive of ectopic pregnancy include an empty uterus with a serum β-hCG level greater than 1500 mUI/mL, cystic or solid adnexal or tubal masses (including the adnexal ring sign, representing a tubal gestational sac), and echogenic or sonolucent cul-de-sac fluid [95]. Agarwal suggests performing a TVUS between the third and fourth week after IVF-ET in all patients who have previously undergone salpingectomy, particularly when β-hCG levels increase slowly [18].

#### 4.3.2. Imaging: MRI and CT

The diagnosis of interstitial ectopic pregnancy may be confirmed via MRI by the presence of an uninterrupted junctional zone separating the gestational sac from the endometrium [96]. MRI may also help differentiate an interstitial or tubal stump ectopic pregnancy from an IUP in an anomalous uterus, such as a bicornuate uterus, allowing the distinction between cornual EP and tubal stump EP. MRI is indicated in the diagnosis of EP when TVUS has a limited role due to factors such as severe tenderness, body habitus, bowel gas, coexistence of ovarian mass, and limited operator experience [97]. In select cases where TVUSs are inconclusive, with suspicion of pregnancy of unknown location (no sac identified on TVUS) in differential diagnosis with other non-gynecologic emergencies (acute appendicitis and acute cholecystitis), MRI is indicated to diagnose EP and localize the pregnancy. Moreover, MRI helps to demonstrate the interstitial or intramural location of EP. Although CT carries the risk of ionizing radiation to the fetus (biologic dose is as high at 35 mSv), on CT, a rounded ring-enhancing mass can be seen abutting the uterine fundus in the cornual region. The myometrium may sometimes be seen to partially surround the gestational sac [98]. Although CT and MRI are not commonly used in the imaging of patients with a positive β-hCG test, various types of ectopic pregnancy are occasionally managed with these modalities. Radiologists should always consider ectopic pregnancy in the setting of hemoperitoneum or a pelvic mass in a woman of childbearing age [99]. The need for laparoscopy when diagnosing ectopic pregnancies has been reduced because of the increased use of TVUS and quantitative measurement of β-hCG [3]. Given the high risk of heterotopic pregnancy, endometrial sampling methods are not recommended (dilation and curettage, frozen section, Karman aspiration, Pipelle sampling) [100,101,102]. Activin-AB and pregnancy-associated plasma protein A were not measured in any of the included studies. Refaat and Bahathiq reported for Activin-AB Se 92.5% and Sp 85%, while in Zhang and Wang, PAPP-A presented a sensitivity of 92.13% and a specificity of 78.33% [103,104]. In a patient with a history of a previous ectopic who presents with all signs of an acute abdomen with severe pain, signs of significant blood loss, combined with a positive urine pregnancy test and an ultrasound examination suggestive of an adnexal mass with gross hemoperitoneum, the first diagnosis to be assumed must be an EP. However, a similar picture can also be associated with early IUP and a bleeding or ruptured corpus luteum. It may also be observed in an early miscarriage and bleeding from another source if there is a history of trauma. One should also consider other causes of abdominal pain, such as acute appendicitis, severe urinary tract infection (UTI), renal colic, ureteric calculus, torsion of the ovary containing the corpus luteum, torsion of an ovarian cyst, and acute pelvic inflammatory disease, to name a few. Any of these may be present with or without a pregnancy.

### 4.4. Treatment

The therapeutic objective is to interrupt the evolution of the ectopic pregnancy and preserve the intrauterine pregnancy. The therapeutic options are numerous [15,52]. The treatment options for EP include surgical and conservative treatments [3,105]. The options for the treatment of interstitial pregnancy combined with intrauterine pregnancy include cornuostomy [40,106], cornual resection [62,87], medical treatment [107], and expectant management [83]. A therapeutic dilemma occurs when the interstitial pregnancy coexists with an intrauterine fetus. In early unruptured interstitial heterotopic pregnancy, local medical treatment with methotrexate or potassium chloride can be used to interrupt it. Patients choosing the expectant management or medical treatment should be informed of the risk of EP rupture, and repeated ultrasound testing should be performed weekly. The suspicion of ectopic pregnancy rupture is an indication to perform surgery with primarily diagnostic purposes. In cases of unruptured pregnancy, in asymptomatic patients without signs of rupture of the ectopic pregnancy, a nonsurgical management is an efficient alternative with a good IUP prognosis [83]. The most frequently described treatment is laparotomic and laparoscopic tubal stump excision. Laparotomy is reserved for cases with life-threatening hemoperitoneum. In Marcus et al. intrauterine pregnancy after surgery proceeds in 50% of cases [108]; in Louis-Sylvestre, heterotopic pregnancies after laparoscopic treatment have a favorable outcome in 62.5% of cases [87]. The second option is medical treatment by ultrasound-guided injection of potassium chloride in situ, rarely combined with methotrexate because of the toxicity of the latter. Although effective, it can lead to persistent trophoblastic tissue.

#### 4.4.1. Expectant Management

Expectant management, combined with ultrasound surveillance, has rarely been found as a therapeutic option due to uncertainty about appropriate patient selection, the efficacy and safety of an expectant approach, and medicolegal concerns because of limited evidence to support expectant management [83,105]. Expectant management may be appropriate for selected patients with early asymptomatic ectopic pregnancies with tubal pregnancy spontaneous resolution ranging from 30% to 70% [109,110,111,112,113]. The effectiveness of the methodology was correlated with β-hCG values, reaching spontaneous resolution in 96% of cases with an initial β-hCG < 175 IU/L [109]. Instead, in patients with an initial β-hCG > 2000 IU/L, the expectant management failed in 93.3% of these cases [112]. A cut-off value of 1000 UI/L for initial β-hCG was shown to offer an acceptable trade-off between sensitivity and specificity for spontaneous resolution [113]. Consider an expectant management, in case of EP, if the initial β-hCG value is <1000 IU/L, with asymptomatic patient and no fetal heart rate and free fluid at TVUS [105]. In case of heterotopic pregnancy with intrauterine pregnancy, the β-hCG criteria cannot be taken into consideration. Sentilhes reports a heterotopic pregnancy, treated with expectant management, because of the absence of fetal heartbeat, the CRL (14 mm), the tubal stump mass (33 mm) and the absence of symptoms, whereas no data regarding β-HCG are reported. The patient weekly repeated clinical and ultrasonographic examinations, demonstrating a gradual involution of the gestational sac with a complete resolution at 20 weeks of pregnancy with an uneventful continuation of the pregnancy. The labor was spontaneous, interrupted by uterine rupture, which manifested itself as dilation stopping at 9 cm, and required a cesarean section. A healthy 4170 g male neonate was delivered [71]. Fernandez treated three heterotopic pregnancies with expectant management, resulting in two full-term deliveries and one failure with subsequent salpingectomy 10 days later for pain and abortion of twin pregnancy at 23 weeks gestation [83].

#### 4.4.2. Surgery

Surgical management was performed in the vast majority of HP cases, reported as 78–90.78% in literature [114,115] and 93.1% in our report. Regarding ampullary and isthmic EP, salpingectomy would not increase uterine rupture risk during pregnancy. Since cornual resection requires myometrial excision, surgery of interstitial EP can lead to an increased risk of IUP uterine rupture in pregnancy [116]. It is unclear whether cornual resection, compared with total tubal resection, can prevent the recurrence of tubal stump ectopic pregnancy. Although there are some cases reported in the literature, there are no studies in this regard [117,118,119,120,121,122,123,124]. Several authors propose performing a purse-string suture technique at the time of cornuostomy to minimize bleeding and extrude the ectopic pregnancy, safely closing the damaged tube or residual stump, both in laparotomy and in laparoscopy [32,125,126]. Moreover, the embedding of the suture into the uterine serosa prevents slipping of the ligature that could occur with a pretied loop [127]. In our cohort, out of 13 patients who underwent cornuostomy, 6 of these were heterotopic pregnancies, all of which reached full term, with delivery via cesarean section. Of these, four cornuostomies were performed by laparotomy, one of these after a failed attempt at medical treatment using intracardiac potassium chloride injection and another two by laparoscopy [106]. Cornuostomy requires follow-up of β-hCG after surgery [105].

Gao et al. report cases of tubal stump ectopic pregnancy after IVF-ET and spontaneous pregnancy. In Gao the laparoscopic approach was used to treat 78.3% of patients with history of previous salpingectomy. Laparoscopic cornuostomy was carried out in 38 cases (82.6%), where 12 had fetal cardiac activity, 15 had ruptured ectopic pregnancy, and 16 used prophylactic methotrexate (MTX) intraoperatively. Median size of the ectopic mass was 2.5 cm (1.0–5.0 cm) [40]. Sun et al. describe 42 cases of tubal stump ectopic pregnancies after IVF-ET and spontaneous pregnancy in patients with history of previous salpingectomy (unilateral 27, bilateral 15 patients). In this study, 22 of 42 (28.6%) tubal stump pregnancies had ruptured ectopic pregnancy at the time of operation. Patients had mean gravidity = 2 (median range 0–4). Similar results concern the cause of previous salpingectomy: previous ectopic pregnancy 71.4% (our study 61.4%) and hydrosalpinx 26.2% (our study 37.5%). In Sun et al. the preoperative β-hCG were IU/L 14,792 (420–77,775) [41].

Kalampokas et al. describe a laparoscopic approach for uterine cornual resection through a step-by-step technique, suggesting the injection of argipressin (1 mL of solution diluted in 100 mL of natural saline 0.9%) immediately close to the area of ectopic pregnancy [59]. Similarly, Balafoutas proposes the key steps of laparoscopic surgery for heterotopic pregnancy, without interfering with the vital intrauterine pregnancy. The first step is to apply a continuous absorbable monofilament suture to the uterine horn around the tubal stump to achieve hemostasis and exposure of the proximal part of the tube, remove the ectopic pregnancy, and close the excision site with a continuous absorbable polyfilament suture [45]. The demonstrated laparoscopic technique is a feasible method to remove the tubal stump pregnancy without interfering with the vital intrauterine pregnancy. Blood loss can be minimized and laparotomy can be avoided. Other authors use vasopressin before tubal stump excision [64,128,129]. Contrary to other authors, Balafoutas advises against the instillation of vasoconstrictive substances and the use of electrical coagulation [45]. It is important to avoid removal of healthy uterine muscle to prevent uterine rupture during future pregnancies. For interstitial laparoscopic pregnancy, Qin et al. recommend performing cornual resection if the interstitial pregnancy portion is bigger than 4 cm with a laparoscopic loop ligation technique, which consists of setting a ring below the distal part of the tube and removing the ectopic pregnancy completely in order to avoid its persistency [130]. Choi et al. propose laparoscopic cornuostomy using a technique similar to that of Kalampokas, using a temporary tourniquet suture and the injection of diluted vasopressin around the cornual mass. The tourniquet suture is removed completely after repairing the cornu [128]. We recommend the use of vasopressin and the ‘purse’ sutures around the ectopic pregnancy to control bleeding. Contraindications to laparoscopic surgery are evidence of internal hemorrhage, shock, or known ruptured ectopic gestation. In case of an ectopic pregnancy on a tubal stump, the only advantages of performing a cornuostomy, compared with the excision of the tubal stump, is to avoid removing uterine muscle tissue, thus protecting against uterine rupture. If surgery is indicated and the surgeon is experienced, laparoscopy is safe and effective and should be the preferred method to manage ectopic pregnancies [131]. However, the included studies do not allow us to establish whether laparoscopy protects against IUP loss in HP with tubal stump EP. Other work has previously associated abortion with laparotomy when compared with laparoscopy [107]. It could be a selection bias, given that laparotomy is more suitable in the presence of factors that can put the life of the fetus and the mother at risk. Chen in 2022 [132] made the comparison of laparoscopic and an open approach in the treatment of heterotopic pregnancy following embryo transfer, evaluating surgical and pregnancy outcomes. Although patients treated by the laparoscopic approach had much less blood loss and fewer days of hospital stay, the rates of first trimester miscarriage, preterm, cesarean section, birth weight, and 1 and 5 min Apgar score were similar between the LA and OA group (all *p* > 0.05) [133]. In Louis-Sylvestre the treatment was surgical in every case and performed laparoscopically in 77% of cases. Ten patients underwent salpingectomy and three underwent salpingostomy. In all cases, 60% of intrauterine pregnancies that were viable at the time of diagnosis of the heterotopic pregnancy had a favorable outcome. Laparoscopy outcome of the intrauterine pregnancy is comparable with that obtained with laparotomy. In three cases, the IUP was non-viable at the time of diagnosis of HP. Of the ten remaining patients, three miscarried within 2 weeks of surgery and one had in utero fetal death of twins after developing chorioamnionitis at 26 weeks. Six women had an uneventful pregnancy (46%) [87]. Even our study did not show a difference in intrauterine outcome between the laparoscopic and laparotomic approach.

#### 4.4.3. Medical Management

Medical treatment could provide different effective choices. These consist of systemic treatment with methotrexate, aspiration, or a combination of aspiration with injections of methotrexate (MTX) or potassium chloride (KCL) in the gestational sac or fetal heart [83,134,135]. Suction and local medical treatment appear to be a feasible option in heterotopic pregnancy with interstitial or cervical pregnancy [88]. In the absence of ruptured pregnancy symptoms, hysteroscopic removal under sonographic guidance after methotrexate treatment or hysteroscopic potassium chloride injection are other conservative methods for managing an interstitial ectopic pregnancy [136]. Transvaginal sonographically guided intracardiac potassium chloride injection is achieved by inserting a needle, directed through the thick myometrium located under the monochorionic sac, to decrease the risk of rupturing the thin myometrium of the cornua [53], according to the technique that was previously described by Timor-Trisch et al. [78]. In heterotopic pregnancies, intracardiac injection of KCl has generally been preferred over MTX because of its potential embryotoxicity. However, this is often associated with slow regression of the trophoblast that could last up to a year due to local trophoblastic invasion of persistent ectopic pregnancy (PEP) [83,137,138,139]. In TVUS the EP is visible as a bulging lesion with a chorionic sac containing the placenta and the live embryo surrounded by a thin myometrial layer. The cavity line does not communicate with this lesion. The probe is rotated into a position with the directional puncture lines to transect the thick uterine myometrium before reaching the gestational sac. Only ectopic pregnancies with positive heartbeats were considered for puncture. A 21-gauge needle is introduced into the chorionic sac and the embryo. The embryo is injected with methotrexate (25 or 50 mg in 1 or 2 mL of solvent, respectively) or with 1 mL of potassium chloride [140]. Since the first report of sonographically guided local injection of KCl for the treatment of heterotopic pregnancy, a handful of cases have been reported in the literature, and some of these regard heterotopic pregnancies with ectopic pregnancy on tubal stump [44,53,84,106,134,138,139,140,141,142]. Benifla reported that when using KCl to treat three interstitial pregnancies in heterotopic pregnancy, only one intrauterine pregnancy was carried to term [139]. Fernandez treated three heterotopic pregnancies with KCl injection in the ectopic sac, resulting in two miscarriages and one intrauterine pregnancy delivered at term [83]. KCl injection protocols used in the literature involve entering the sac, aspiration of the celomic fluid, and KCl injection directly into the fetal thorax until the observation of the cessation of fetal heart movements. The whole procedure lasts 15 min. It could be performed without analgesia or anesthesia and had no immediate or late complications [83,134,138,139]. MTX is a folic acid antagonist that inhibits dihydrofolate reductase, resulting in impaired DNA synthesis in rapidly dividing cells. The systemic MTX protocol for EP, particularly if ectopic mass is less than 4 cm, was adapted from protocols designed for gestational trophoblastic disease and uses a fixed multidose schedule in which methotrexate (50 mg/m^2^) is given via intramuscular (IM) injection in a single IM dose or two IM doses, with similar effectiveness and less costs compared with surgery, the gold standard treatment for EP [143,144]. As an alternative to local or systemic intramuscular administration, oral administration is also possible with an overall success rate of 80% [78,145,146]. Bernardini does not recommend local treatment of EP, preferring the systemic one [144]. Potential risks deriving from the local administration of MTX are ectopic pregnancy rupture and bleeding.

In case of HP, the use of systemic MTX is contraindicated because of its embryotoxicity on the IUP, but recent studies have proved its safety and effectiveness with embryo suction combined with local injection of low-dose MTX [147,148]. Failure of methotrexate therapy or intracardiac potassium chloride injection must be considered; close follow-up is required and may lead to surgery with cornuostomy or tubal stump excision [38]. A procedure of local aspiration combined with instillation of MTX in a heterotopic pregnancy is described in Oyawoye et al. The use of MTX can lead to the prevention of local trophoblastic invasion, which is likely to occur with the use of KCl, and avoidance of the possible risk of cornual rupture, especially in the presence of an ongoing intrauterine pregnancy. The aspiration occurs under ultrasound guidance followed by local injection of 12.5 mg of methotrexate into the cavity using a 16-gauge double lumen ovum pickup needle. A regulated vacuum pump is used at a negative pressure of 230 mm/Hg. The fetal pole in the ectopic pregnancy disappears following the procedure. The whole procedure including the initial preparations lasts 10 min and is less expensive than surgery. It is important to advise taking 5 mg of folic acid every day. Oyawoye describes the technique in a case of HP that continued in full-term pregnancy resulting in vaginal delivery without any complications [149].

Qiong et al. describe selective reduction by aspiration of the fetal heart of the ectopic pregnancy under transvaginal ultrasonographic guidance at 4 to 6 weeks after embryo transfer, performed in five patients with heterotopic pregnancy. An introducer needle is attached to a probe, through which the 17-gauge aspirating needle is later introduced through the vaginal fornix until it reaches the gestation sac and the fetal heart until aspiration of the fetal heart along with products of conception, regulating the vacuum pump at 220 mm/Hg. The patients were subsequently followed up ultrasonographically on postoperative days 2 and 7 to verify asystole of the aspirated fetus and no further growth of the ectopic pregnancy. None of the five patients who underwent selective ectopic embryo reduction required further treatment. All had no complications. However, in one patient, an early spontaneous abortion occurred on postoperative day 4, and in another, a late spontaneous abortion occurred 3 months postoperatively. The remaining three patients delivered healthy babies [150].

Based on the data collected, the gestational age of delivery of intrauterine pregnancy is independent from the ectopic pregnancy event and the treatment but mainly depends on the type of intrauterine pregnancy, singleton or twin.

### 4.5. Clinical Recommendations

Based on the evidence reviewed, we propose the following clinical recommendations for the management of patients with prior salpingectomy undergoing IVF-ET:

- Early and serial monitoring: Given the high risk of recurrence, all patients with prior salpingectomy undergoing IVF-ET should undergo an early transvaginal ultrasound (TVUS) at 6 weeks of gestation, combined with serial β-hCG monitoring.
- Symptom vigilance: A close follow-up is recommended until the 8th–10th week of pregnancy, with particular attention to abdominal pain, which is the most frequent symptom of tubal stump EP.
- High index of suspicion: Clinicians should maintain a high index of suspicion for heterotopic pregnancy, even in the presence of a visualized intrauterine sac, especially in women with bilateral salpingectomy.
- Individualized treatment: Management (expectant, medical, or surgical) should be guided by the hemodynamic stability of the patient, the presence of fetal heartbeat in the ectopic sac, and the desire to preserve the concomitant intrauterine pregnancy.

### 4.6. Strengths and Limitations

The protocol is reported according to the Preferred Reporting Items for Systematic Reviews and Meta-Analysis (PRISMA) Guidelines 2020 [24]. The proposed review was conducted by applying the latest Cochrane recommendations and tools for systematic reviews. The protocol incorporated a comprehensive search strategy to include all eligible studies that matched the inclusion criteria, with no language or publication date restrictions. The screening, selection, data extraction, and quality assessment were conducted by two reviewers independently. Any discrepancies were resolved by consensus or consultations with a third reviewer.

However, several limitations must be acknowledged. First, the completeness of the review is limited by heterogeneity in data reporting and lack of data for several studies. Second, technical details of the embryo transfer procedure (e.g., catheter placement depth, fluid volume) were largely unavailable in the primary reports, preventing an analysis of procedural risk factors. Third, although the electronic search strategy was designed by experienced content and methods experts, it was not formally peer-reviewed according to the PRESS guideline, which may have left some residual room for suboptimal sensitivity or specificity of the searches. Fourth, the literature search was completed on 30 April 2024; while this date represents the pre-specified cut-off in our protocol, the time elapsed between the search and publication may be a limitation. Finally, the vast majority of the included studies are case reports or small case series. This introduces a significant risk of publication bias (extreme or atypical cases are more likely to be published) and limits the statistical independence of the observations. Therefore, the quantitative analyses presented should be interpreted as exploratory and descriptive of the published literature rather than as robust inferential evidence generalizable to all clinical settings.

## 5. Conclusions

This is the first paper that can be used for adequate counseling with a patient presenting a heterotopic pregnancy with tubal stump ectopic pregnancy. Heterotopic pregnancy occurs in 60% of cases of EP after IVF-ET and bilateral salpingectomy; of these, 82.7% are singletons and 15.2% are twins. In 81.9% of cases the outcome of intrauterine pregnancy was delivery. Delivery at term occurred in 95.5% of singleton intrauterine pregnancies. In patients with a positive β-hCG without the finding of an intrauterine developing pregnancy at the ultrasound scan, a careful follow-up must be arranged with the goal of performing a definitive diagnosis of an IUP, ectopic pregnancy, or persistent PUL. Although the heterotopic pregnancy after IVF-ET remains a rare occurrence, we argue that a close follow-up is useful until the 8th–10th week of pregnancy considering a gestational age IQR at diagnosis of tubal stump ectopic pregnancy: 35–56 days in the case of IVF-ET with positive beta-hcg dosage; furthermore, the patient must monitor the symptoms, particularly abdominal pain, which is the most frequent symptom. Ectopic pregnancy rupture does not influence the obstetric outcome of intrauterine pregnancy without increasing in miscarriage, nor does it change the gestational age at delivery. The most common surgical management chosen is tubal stump excision (74.1%), followed by cornuostomy (24.1%). The classification of ectopic pregnancies should consider those obtained after surgery, for example after adnexectomy or salpingectomy, determining sites of ectopic pregnancy that otherwise, naturally, would not exist. Successful treatment of ectopic pregnancy may be expectant, medical, or surgical, or it may involve a combination of several approaches, depending on initial β-hCG level, ultrasound findings, and patient characteristics; medical treatment is an area of research worth evaluating and has encouraging obstetric outcomes. The median (IQR) intervals between salpingectomy and occurrence of ipsilateral (or bilateral) EP were 36 months in the case of unilateral salpingectomy and 12 in the case of bilateral salpingectomy. The time elapsed from surgery to diagnosis of ectopic pregnancy may be relevant. Perhaps tissue inflammation and early scarring phenomena could predispose to the onset of ectopic pregnancy.

## Figures and Tables

**Table 1:** Main characteristics of included studies.

Author	Year	Country	Publication Type	No. of Cases	HP at Least in One of the Cases	Treatment
Al-sunaidi [42]	2007	Canada	Case report	1	No	Laparotomy
Arbab [43]	1996	France	Case report	2	Yes	Laparotomy
Baker [44]	1997	United States	Case report	1	Yes	Medical management
Balafoutas [45]	2021	Germany	Video Article	1	No	Laparoscopy
Banzai [46]	2021	Japan	Case report	1	Yes	Laparoscopy
Ben-ami [47]	2006	Israel	Case report	1	Yes	Laparoscopy
Bhat [48]	2004	Oman	Case report	1	Yes	Laparotomy
Blazar [49]	2007	United States	Case report	1	Yes	Laparotomy
Bornstein [50]	2011	United States	Letter to editor	1	Yes	Laparotomy
Chang [14]	2003	Taiwan	Case report	1	Yes	Laparotomy
Chen [51]	1998	Taiwan	Case report	2	No	Laparotomy
Chin [52]	2004	Taiwan	Case report	2	Yes	Laparotomy
Van der Weiden [53]	2001	The Netherlands	Letter to editor	1	Yes	Medical management
Divry [54]	2007	France	Case report	1	Yes	Laparotomy
Dumesic [55]	2001	United States	Case report	1	Yes	Laparotomy
Felemban [56]	2018	Saudi Arabia	Case report	1	No	Laparoscopy
Garavaglia [57]	2012	Italy	Case report	2	No	Laparoscopy
Ji [58]	2019	China	Case report	1	Yes	Laparoscopy
Kalampokas [59]	2022	Greece	Case report	1	No	Laparoscopy
Kasum [15]	1998	Croatia	Case report	1	Yes	Laparotomy
Khoo [60]	2014	Singapore	Case report	1	Yes	Laparoscopy
Ko [7]	2011	Taiwan	Case series (consecutive)	4	Yes	Laparoscopy
Lower [61]	1989	United Kingdom	Case report	1	Yes	Laparotomy
Lund [62]	1989	United States	Case report	1	No	Laparotomy
Manea [63]	2014	Switzerland	Case report	1	No	Laparoscopy
Maruthini [64]	2013	United Kingdom	Case report	2	No	Laparoscopy
Melcer [65]	2021	Israel	Case control	5	Yes	Laparoscopy
Okamura [66]	2011	Japan	Case report	1	Yes	Laparoscopy
Oral [67]	2014	Turkey	Case report	1	Yes	Laparoscopy
Pan [68]	2010	China	Case report	1	No	Laparotomy
Pavic [69]	1986	Switzerland	Case report	1	No	Laparotomy
Piccioni [38]	2017	Italy	Case report	2	No	Laparoscopy
Prorocic [70]	2012	Serbia	Case report	1	Yes	Medical management
Sentilhes [71]	2009	France	Case report	1	Yes	Expectant management
Sharif [72]	1994	United Kingdom	Case report	1	Yes	Laparotomy
Shavit [73]	2013	Israel	Case report	2	Yes	Laparoscopy
Sills [74]	2002	United States	Case report	1	Yes	Laparotomy
Wang [75]	2021	China	Case report	4	Yes	Laparoscopy
Xi [76]	2019	China	Case report	2	Yes	Laparotomy
Yip [77]	2020	Singapore	Case report	1	No	Laparoscopy

**Table 2:** Study population.

Age, years (mean ± SD)		32.6 (3.7)
Gravidity, median (IQR)		2 (1–3)
Parity, median (IQR)		0 (0–0)
Nulliparous	no. (%)	39 (76.5)
Gravidity ≥ 1	no. (%)	45 (90)
Multiparous	no. (%)	2 (3.9)
**Type of fallopian tube resection**		
Laparoscopic	no. (%)	26 (44.8)
Laparotomic	no. (%)	5 (8.6)
Not specified	no. (%)	27 (46.6)
**Side of salpingectomy**		
Right	no. (%)	10 (17.2)
Left	no. (%)	8 (13.8)
Bilateral	no. (%)	40 (69.0)
**Cause of fallopian resection on side of pregnancy**		
Hydrosalpinx	no. (%)	21/56 (37.5)
PID	no. (%)	2
Preparation to IVF-ET	no. (%)	3
Tubal ectopic pregnancy	no. (%)	35/57 (61.4)
**If previous tubal EP:**		
No. of previous TUBAL EP due to SPONTANEOUS pregnancy, median (IQR)		0 (0–1)
No. of previous TUBAL EP due to IVF-ET, median (IQR)		0 (0–1)

SD: standard deviation; IQR: interquartile range; EP: ectopic pregnancy.

**Table 3:** Characteristics of IVF-ET and EP.

**IVF-ET and EP**		
Median (IQR) no. of transferred embryo (n = 40)		2 (2–3)
Type of embryo transfer, n (%)	Not specified	21/40 (52.5)
	Fresh	10/40 (25.0)
	Frozen	8/40 (20.0)
	Fresh-frozen	1/40 (2.5)
Median (IQR) no. of previous ET, cycles (n = 24)		1.5 (1–3)
Median (IQR) GA at diagnosis, days (n = 56)		45.5 (35–56)
**US characteristics**		
Visible fetal heartbeat no. (%)	No	7/25 (28.0)
	Yes	18/25 (72.0)
Median (IQR) stump large diameter, mm (n = 9)		25 (20–33)
Median (IQR) gestational sac, mm (n = 9)		14 (11–17)
Median (IQR) CRL, mm (n = 9)		8.8 (6–14)
**Symptoms** no. (%)		
Vaginal bleeding	No	35/51 (68.6)
	Yes	16/51 (31.4)
Abdominal pain	No	15/53 (28.3)
	Yes	38/53 (71.7)
Hypovolemic shock symptoms	No	39/51 (76.5)
	Yes	12/51 (23.5)
Internal bleeding no. (%)		40/58 (69)
Median (IQR) internal bleeding, mL (n = 28)		650 (200–1350)
**Preoperative parameters**		
Median (IQR) Hb, gr/dL (n = 14)		8.5 (7.5–11.5)
Median (IQR) systolic blood pressure, mmHg (n = 13)		100 (90–118)
Median (IQR) diastolic blood pressure, mmHg (n = 12)		65 (51.0–74.5)
Median (IQR) pulse rate, bpm (n = 9)		100 (95–120)
Median (IQR) units of red blood cells (n = 9)		3 (2–4)
Median (IQR) length of stay, day (n = 15)		4 (1–7)

IQR: interquartile range; EP: ectopic pregnancy; CRL: crown rump length; Hb: Hemoglobin.

**Table 4:** Characteristics of ectopic pregnancy and ruptured pregnancy: gestational age at diagnosis, symptoms, laboratory, ultrasound findings.

		Rupture Pregnancy		*p*-Value
		No	Yes	
Ruptured pregnancy, n (%)		17/56 (30.4)	39/56 (69.6)	
Median (IQR) gestational age at diagnosis, days (n = 17 vs. 37)		48 (37–56)	49 (35–56)	0.96
Hypovolemic shock symptoms, n (%)		0/16 (0.0)	12/35 (34.3)	0.01
Vaginal bleeding, n (%)		7/16 (43.8)	9/35 (25.7)	0.20
Surgical procedure, n (%)	Laparoscopy	7/17 (41.2)	21/39 (53.9)	0.16
	Laparotomy	7/17 (41.2)	17/39 (43.6)	
	Medical treatment	3/17 (17.7)	1/39 (2.6)	
Heartbeat, n (%)		11/12 (91.7)	7/13 (53.9)	0.07
Abortion, n (%)		1/10 (10.0)	4/22 (18.2)	1.00
Delivery at term, n (%)		7/9 (77.8)	16/18 (88.9)	0.58
Median (IQR) preoperative β-HCG in non-heterotopic pregnancy (n = 4 vs. 5)		28,881 (17,768–55,379)	4960 (2293–8839)	0.02
Median (IQR) stump larger diameter, mm (n = 3 vs. 6)		18 (15–33)	25 (22–55)	0.21

Chi-square test for categorical variables and the Mann–Whitney test for quantitative variables. IQR: interquartile range.

**Table 5:** HP characteristics and pregnancy outcome.

HP, n (%)		33/50 (60.0)
If HP: type of intrauterine pregnancy, n (%)	Singleton	28/33 (82.7)
	Twin	5/33 (15.2)
If HP: outcome of intrauterine pregnancy, n (%)	Caesarean section	22/33 (66.7)
	Abortion	6/33 (18.2)
	Delivered at term (not specified)	3/33 (9.1)
	Vaginal delivery	2/33 (6.1)
Caesarean section, n (%)	Elective	12/22 (54.6)
	Emergency	7/22 (31.8)
	Non specified	3/22 (13.6)
Delivery at term, n (%)		23/27 (85.2)
Healthy baby, n.	Yes	25/27
	Not specified	2/27
Median (IQR) birth weight, g (n = 4)		2365 (1915–2814)

IQR: interquartile range; HP: heterotopic pregnancy.

**Table 6:** Management of ectopic pregnancy.

Surgical procedure, n (%)	Laparoscopy	30/58 (51.7)
	Laparotomy	24/58 (41.4)
	Medical treatment	4/58(6.9)
If laparotomy: conversion from laparoscopy, n (%)		7/15 (46.7)
Surgical management, n (%)	Tubal stump excision	40/54 (74.1)
	Cornuostomy	13/54 (24.1)
	Hysterectomy	1/54 (1.9)

**Table 7:** Treatment and IU pregnancy outcome.

	**Laparoscopy**	**Laparotomy**	**Medical Treatment**	***p*** **-Value**
Abortion, n (%)	3/15 (20.0)	3/14 (21.4)	0/4 (0.0)	1.00
Delivery at term, n (%)	11/12 (91.7)	8/11 (72.7)	4/4 (100.0)	0.34
	**Laparoscopy + Laparotomy**		**Medical treatment**	***p*** **-value**
Abortion, n (%)	6/29 (20.7)		0/4 (0.0)	1.00
Delivery at term, n (%)	19/23 (82.6)		4/4 (100.0)	1.00

Chi-square test for categorical variables and the Mann–Whitney test for quantitative variables. IU: intrauterine.

**Table 8:** Length of stay stratified by surgical procedure and months interval from salpingectomy to the occurrence of EP.

	**Laparoscopy**	**Laparotomy**	***p*** **-Value**
Median (IQR) length of postoperative hospitalization, day (n = 6 vs. 8)	1.5 (1–3)	6 (4.5–7.0)	0.06
	**Unilateral salpingectomy**	**Bilateral salpingectomy**	***p*** **-value**
Median (IQR) interval between salpingectomy and occurrence of ipsilateral (or bilateral) EP, months (n = 7 vs. 17)	36 (12–84)	12 (12–24)	0.17

Chi-square test for categorical variables and the Mann–Whitney test for quantitative variables. Comparison of median (IQR) months interval from salpingectomy to the occurrence of ipsilateral (or bilateral) EP by type of salpingectomy. Chi-square test for categorical variables and the Mann–Whitney test for quantitative variables. IQR: interquartile range; EP: ectopic pregnancy.

**Table 9:** Findings certainty assessment via the GRADE CER-Qual assessment.

Finding no.	Review Finding Groups	Outcomes	No. of Studies	No. of Participants	*p*-Value	Certainty of the Evidence (GRADE-CERqual)	GRADE-CERqual Assessment	Comment
1	1. Demographic information and gynecological history	mean (±SD) age of patients 32.6 (±3.7) years.	40	58	-	⊕⊕⊕⊕ High confidence	-	This condition affects women between young adulthood (YA) and adulthood.
2		90% of the patients had gravidity ≥ 1.	35	50	-	⊕⊕⊕⊝ Moderate confidence	Although the literature suggests including the current pregnancy when indicating gravidity, some authors did not include it. Clarifications were requested from the authors; we did not obtain the requested clarifications. We have tried, where possible, to standardize the gravidity value without considering the current pregnancy.	Most patients with ectopic pregnancies on tubal stumps have had a high gravidity rate.
3		76.5% of the patients had parity = 0.	36	51	-	⊕⊕⊕⊕ High confidence	-	Although the majority of patients with ectopic pregnancies on tubal stumps have had a high gravidity rate, the majority of them have not had pregnancies.
4		73.4% of patients had previously had 1 to 5 ectopic pregnancies.	34	49	-	⊕⊕⊕⊕ High confidence	-	Most patients with an EP on a tubal stump had had at least one ectopic pregnancy prior to salpingectomy.
5		The cause of previous salpingectomy: previous tubal pregnancy 61.4%, hydrosalpinx 37.5%. Two patients had previous PID.	39	57	-	⊕⊕⊕⊕ High confidence	-	The most frequent cause of previous salpingectomy in patients with tubal stump ectopic pregnancy was previous tubal pregnancy.
6		Median interval between salpingectomy and occurrence of ipsilateral (or bilateral) EP varies between 36 months (IQR: 15.0–84.0 months) after unilateral salpingectomy and 12 months (IQR:12–24.0 months) after bilateral salpingectomy.	11	24	0.17	⊕⊕⊝⊝ Low confidence	The data are provided differently among the studies, in some cases expressed in months, in other cases in years. We chose “months” as a unit of measurement; when the number of months were not specified, we considered what was indicated (e.g., 1 year = 12 months).	There may be a difference in the latency between salpingectomy and occurrence of PE in unilateral salpingectomy compared with bilateral salpingectomy. The data available are few and heterogeneous. More data are needed.
7	2. IVF-ET	69.0% of patients had undergone bilateral salpingectomy. No significant difference in EP side was reported between right 18/40 (45%) and left 22/40 (55.0%) side. Only 1 patient presented bilateral ectopic pregnancy.	30	40	-	⊕⊕⊕⊕ High confidence	-	Most patients with tubal stump ectopic pregnancy had undergone bilateral salpingectomy. In this population, there was no reported significant difference in EP side.
8		Median of transferred embryo was 2 (IQR 2–3).	34	40	-	⊕⊕⊝⊝ Low confidence	The number of transferred embryos is not specified in type of embryos, developmental stage, technique used. A stratification would have been useful, but there would not have been an adequate amount of data.	Tubal stump ectopic pregnancy can occur with intrauterine transfer of 2 embryos
9		Fresh embryo was 25.0%, frozen 20.0%, not specified 52.5%.	15	35	-	⊕⊕⊝⊝ Low confidence	Are not specified in type of embryos, developmental stage, technique used. A stratification would have been useful, but there would not have been an adequate amount of data.	Tubal stump ectopic pregnancy can occur with intrauterine transfer of fresh embryos and frozen embryos.
10	EP and Pregnancy	Median (IQR) gestational age at diagnosis was 45.5 (35–56) days.	38	56	-	⊕⊕⊕⊝ Moderate confidence	Heterogeneous data across studies: some provided in days, others in weeks. When the number of days or the number of months were not specified, we considered what was indicated (e.g., 1 year = 12 months, 1 week = 7 days). If explicitly indicated, the exact number of days was considered (e.g., 5 weeks + 3 days = 38 days). Anything marked as “1/2 week” was then rounded down: 3 days.	Gestational age IQR at diagnosis of tubal stump ectopic pregnancy: 35–56 days.
11	Symptoms and clinical findings	Hypovolemic shock symptoms were related to ruptured pregnancy.	37	51	0.01	⊕⊕⊕⊕ High confidence	-	Hypovolemic shock symptoms occur with EP rupture.
12		71.7% of patients reported abdominal pain, making it the most frequent symptom. The others, in order of frequency, were vaginal bleeding (31.4%) and hypovolemic shock symptoms (23.5%). Only 4 patients of 51 had the complete triad (amenorrhea, abdominal pain, and vaginal bleeding).	37	51	-	⊕⊕⊕⊝ Moderate confidence	Not all symptom categories were described in all studies; a small number of data available.	In tubal stump ectopic pregnancy abdominal pain is the symptom most reported.
13	Treatment and intraoperative findings	Expectant and medical management are promising new approaches that need to be further evaluated. With the exception of failures of medical therapy, with the need for surgical treatment, medical therapy has a good outcome of intrauterine pregnancies.	6	6	-	⊕⊝⊝⊝ Very low confidence	There are few studies available regarding the medical treatment of tubal stump ectopic pregnancy. Lack of data.	Expectant and medical management are promising approaches
14		Nearly 46.7% of patients underwent laparotomy following conversion from laparoscopy. Most of the surgical management consisted of tubal stump excision (74.1%), followed by cornuostomy (24.1%). Only in one case was hysterectomy necessary.	40	52	-	⊕⊕⊕⊕ High confidence	-	In tubal stump ectopic pregnancy, tubal stump excision is the most used procedure.
15		Ectopic pregnancy rupture does not influence the obstetric outcome of intrauterine pregnancy: there is no increase in miscarriage.	39	56	1.00	⊕⊕⊕⊕ High confidence	-	Ectopic pregnancy rupture does not influence the obstetric outcome of intrauterine pregnancy.
16		Ectopic pregnancy rupture does not influence the obstetric outcome of intrauterine pregnancy: there is no change in gestational age at delivery.	39	56	0.58	⊕⊕⊕⊕ High confidence	-	
17	Ultrasound appearance	TVUS: 72.0% of cases with evidence of a fetal heartbeat, which was present in 91.7% of cases of pregnancy without rupture and in 53.9% of ruptured pregnancies.	23	25	0.07	⊕⊕⊕⊝ Moderate confidence	Limited amount of data.	The fetal heartbeat is less frequent with a ruptured pregnancy.
18	Heterotopic pregnancy outcome	Heterotopic pregnancy occurs in 60% of cases; of these, 82.7% are singletons and 15.2% are twin. In 81.9% of cases the outcome of intrauterine pregnancy was delivery (cesarean section in 66.7% of cases, 6.1% vaginal delivery, and 9.1% unspecified type of delivery), and 18.2% of intrauterine pregnancy evolves into miscarriage.	27	33	-	⊕⊕⊕⊕ High confidence	-	In case of ectopic pregnancy on tubal stump following IVF-ET, heterotopic pregnancy is common. Most intrauterine pregnancies reach full term with delivery via cesarean section.
19		The caesarean section was elective in 54.6%, while in 31.8% it was performed as an emergency (13.6% is not specified the mode of cesarean section).	18	22	-	⊕⊕⊕⊕ High confidence	-	It may be useful to plan the caesarean section to be performed electively.
20		25 patients of 27 delivered healthy babies; in the remaining two cases it was not specified.	23	27	-	⊕⊕⊕⊕ High confidence	-	Most babies were healthy at birth.
21		Median birth weight is 2365 (IQR: 1915–2814). Singleton birth median weight: 2365 gr (IQR 1915–2814); twin birth median weight: 2490 gr (IQR 1590–2500).	17	18	-	⊕⊕⊕⊕ High confidence	-	Most babies were of adequate weight at birth.
22		3 cases of medical treatment out of 5 were successful. These 3 cases and expectant management presented an intrauterine pregnancy that reached full term.	6	6	-	⊕⊝⊝⊝ Very low confidence	There are few studies available regarding the medical treatment of tubal stump ectopic pregnancy. Lack of data.	Expectant and medical management are promising approaches for efficacy of treatment of ectopic pregnancy and for outcome of intrauterine pregnancy.
23		Delivery at term occurred in 85.2% of all intrauterine pregnancies, of which singleton intrauterine pregnancies delivered at term in 95.5% of cases instead of 40.0% of cases of twin intrauterine pregnancies.	23	27	-	⊕⊕⊕⊕ High confidence	Some studies did not provide the exact week of the gestational age of delivery of the intrauterine pregnancy but only indicated “term”; we considered a cut-off of 37 weeks, obtaining two groups (1st < 37 weeks; 2nd ≥ 37 weeks + term).	Delivery at term of the intrauterine pregnancy occurred in almost all singleton pregnancies.

## Data Availability

The authors confirm that the data underlying this article are available in the article and in its online Supplementary Materials.

## Supplementary Materials

The following supporting information can be downloaded at: https://www.mdpi.com/article/10.3390/medicina62010083/s1, Figure S1: Modified JBI checklist for case report and case series (tool for systematic review) plot; Table S1: Preferred Reporting Items for Systematic Reviews and Meta-Analysis (PRISMA) checklist of items to include when reporting a systematic review and meta-analysis [24]; Table S2: PICO strategy and MeSH terms; Table S3: List of studies excluded after full-text assessment with reasons; Table S4: Index data and collecting data rules [25,26,27,28,29,30,31,32,33,34]; Table S5: Modified JBI checklist for case report and case series (tool for systematic review) [7,14,15,42,43,44,45,46,47,48,49,50,51,52,53,54,55,56,57,58,59,60,61,62,63,64,65,66].

## Abbreviations

The following abbreviations are used in this manuscript:

ARTs	Assisted reproductive technologies
CRL	Crown-rump length
EP	Ectopic pregnancy
GIFT	Gamete intrafallopian transfer
GRADE-CERQual	GRADE-CERQual Interactive Summary of Qualitative Findings
Hb	Hemoglobin
HP	Heterotopic pregnancy
IM	Intramuscular
IQR	Interquartile range
iSoQ	Interactive Summary of Qualitative Findings
IU	Intrauterine
IUD	Intrauterine device
IUP	Intrauterine pregnancy
IVF-ET	In vitro fertilization-embryo transfer
JBI	Joanna Briggs Institute
KCl	Potassium chloride
MTX	Methotrexate
NRSI	Non-randomized studies of interventions
PEP	Persistent ectopic pregnancy
PICO	Population/patients, intervention/exposure, control/comparison, and outcome
PID	Pelvic inflammatory disease
PRISMA	Preferred Reporting Items for Systematic Reviews and Meta-Analysis
PROSPERO	International Prospective Register of Systematic Reviews
PUL	Pregnancy of unknown location
SD	Standard deviation
TVUS	Transvaginal ultrasound
US	Ultrasound
