# Supplementary material for: Tubal Stump Ectopic Pregnancy After IVF-ET in Patients Who Underwent Salpingectomy or Adnexectomy: A Qualitative Systematic Review
Source: Medicina (Kaunas). 2025 Dec 31;62(1):83. doi: 10.3390/medicina62010083 (PMC12843002; doi:10.3390/medicina62010083)
Supplement: Supplementary file 1 [file medicina-62-00083-s001.zip › medicina-4018429-supplementary.pdf]

Table S1. Preferred Reporting Items for Systematic Reviews and Meta-Analysis (PRISMA) Checklist of items to include when reporting a systematic review and meta-analysis (Page et al., 2021).

Section and Topic	Item #	Checklist item	Location where item is reported
TITLE			
Title	1	Identify the report as a systematic review.	1
ABSTRACT			
Abstract	2	See the PRISMA 2020 for Abstracts checklist.	Abstract
INTRODUCTION			
Rationale	3	Describe the rationale for the review in the context of existing knowledge.	Introduction, paragraph 36
Objectives	4	Provide an explicit statement of the objective(s) or question(s) the review addresses.	Introduction, paragraph 38
METHODS			
Eligibility criteria	5	Specify the inclusion and exclusion criteria for the review and how studies were grouped for the syntheses.	Methods, paragraph 5
Information sources	6	Specify all databases, registers, websites, organisations, reference lists and other sources searched or consulted to identify studies. Specify the date when each source was last searched or consulted.	Methods, paragraph 31
Search strategy	7	Present the full search strategies for all databases, registers and websites, including any filters and limits used.	Methods, paragraph 31
Selection process	8	Specify the methods used to decide whether a study met the inclusion criteria of the review, including how many reviewers screened each record and each report retrieved, whether they worked independently, and if applicable, details of automation tools used in the process.	Methods, paragraph 32
Data collection process	9	Specify the methods used to collect data from reports, including how many reviewers collected data from each report, whether they worked independently, any processes for obtaining or confirming data from study investigators, and if applicable, details of automation tools used in the process.	Methods, paragraph 37
Data items	10a	List and define all outcomes for which data were sought. Specify whether all results that were compatible with each outcome domain in each study were sought (e.g. for all measures, time points, analyses), and if not, the methods used to decide which results to collect.	Methods
	10b	List and define all other variables for which data were sought (e.g. participant and intervention characteristics, funding sources). Describe any assumptions made about any missing or unclear information.	Methods, paragraph 20
Study risk of bias assessment	11	Specify the methods used to assess risk of bias in the included studies, including details of the tool(s) used, how many reviewers assessed each study and whether they worked independently, and if applicable, details of automation tools used in the process.	Methods, paragraph 40
Effect measures	12	Specify for each outcome the effect measure(s) (e.g. risk ratio, mean difference) used in the synthesis or presentation of results.	Methods, paragraph 43
Synthesis methods	13a	Describe the processes used to decide which studies were eligible for each synthesis (e.g. tabulating the study intervention characteristics and comparing against the planned groups for each synthesis (item #5)).	Methods
	13b	Describe any methods required to prepare the data for presentation or synthesis, such as handling of missing summary statistics, or data conversions.	Methods
	13c	Describe any methods used to tabulate or visually display results of individual studies and syntheses.	Methods
	13d	Describe any methods used to synthesize results and provide a rationale for the choice(s). If meta-analysis was performed, describe the	Methods,

Section and Topic	Item #	Checklist item	Location where item is reported
		model(s), method(s) to identify the presence and extent of statistical heterogeneity, and software package(s) used.	paragraph 43
	13e	Describe any methods used to explore possible causes of heterogeneity among study results (e.g. subgroup analysis, meta-regression).	n/a
	13f	Describe any sensitivity analyses conducted to assess robustness of the synthesized results.	n/a
Reporting bias assessment	14	Describe any methods used to assess risk of bias due to missing results in a synthesis (arising from reporting biases).	Methods paragraph 61
Certainty assessment	15	Describe any methods used to assess certainty (or confidence) in the body of evidence for an outcome.	Methods paragraph 61
**RESULTS**			
Study selection	16a	Describe the results of the search and selection process, from the number of records identified in the search to the number of studies included in the review, ideally using a flow diagram.	Results, paragraph 1
	16b	Cite studies that might appear to meet the inclusion criteria, but which were excluded, and explain why they were excluded.	Results, paragraph 1
Study characteristics	17	Cite each included study and present its characteristics.	Results, paragraph 9
Risk of bias in studies	18	Present assessments of risk of bias for each included study.	Methods, paragraph 40
Results of individual studies	19	For all outcomes, present, for each study: (a) summary statistics for each group (where appropriate) and (b) an effect estimate and its precision (e.g. confidence/credible interval), ideally using structured tables or plots.	Results, paragraph 9
Results of syntheses	20a	For each synthesis, briefly summarise the characteristics and risk of bias among contributing studies.	Results, paragraph 10
	20b	Present results of all statistical syntheses conducted. If meta-analysis was done, present for each the summary estimate and its precision (e.g. confidence/credible interval) and measures of statistical heterogeneity. If comparing groups, describe the direction of the effect.	Results, paragraph 10
	20c	Present results of all investigations of possible causes of heterogeneity among study results.	Results, paragraph 10
	20d	Present results of all sensitivity analyses conducted to assess the robustness of the synthesized results.	Results, paragraph 10
Reporting biases	21	Present assessments of risk of bias due to missing results (arising from reporting biases) for each synthesis assessed.	Methods paragraph 61
Certainty of evidence	22	Present assessments of certainty (or confidence) in the body of evidence for each outcome assessed.	Supplementary materials (GRADE CERqual)
**DISCUSSION**			
Discussion	23a	Provide a general interpretation of the results in the context of other evidence.	Discussion
	23b	Discuss any limitations of the evidence included in the review.	Conclusions,

Section and Topic	Item #	Checklist item	Location where item is reported
			paragraph 13
	23c	Discuss any limitations of the review processes used.	Conclusions
	23d	Discuss implications of the results for practice, policy, and future research.	Conclusions
OTHER INFORMATION			
Registration and protocol	24a	Provide registration information for the review, including register name and registration number, or state that the review was not registered.	Methods paragraph 7
	24b	Indicate where the review protocol can be accessed, or state that a protocol was not prepared.	Methods paragraph 5
	24c	Describe and explain any amendments to information provided at registration or in the protocol.	Methods paragraph 6
Support	25	Describe sources of financial or non-financial support for the review, and the role of the funders or sponsors in the review.	Funding
Competing interests	26	Declare any competing interests of review authors.	Conflict of interests
Availability of data, code and other materials	27	Report which of the following are publicly available and where they can be found: template data collection forms; data extracted from included studies; data used for all analyses; analytic code; any other materials used in the review.	Supplementary tables

From Page MJ, Mckenzie JE, Bossuyt PM, Boutron I, Hoffmann C, Mulrow CD, Shamseer L, Tetzlaff JM, Akl EA, Brennan SE, et al. The PRISMA 2020 statement: an updated guideline for reporting systematic reviews Systematic reviews and Meta-Analyses. [24]

Table S2. PICO strategy and MeSH terms

	PICO	MeSH terms
POPULATION / PROBLEM	Ectopic pregnancy in tubal stump	Pregnancy trimester, first; pregnancy, ectopic;
INTERVENTION / EXPOSURE	after Salpingectomy or adnexectomy and IVF-ET	salpingectomy; embryo transfer;
CONTROL / COMPARISON INTERVENTION (IF ANY)	Not appliable	Not appliable
OUTCOME(S)	Prevention, diagnosis, treatment, intrauterine pregnancy outcome	Primary prevention; secondary prevention; diagnosis; pregnancy outcome;

Table S3. List of studies excluded after full-text assessment with reasons.

Study (First Author, Year)	Reason for Exclusion
Ouyang et al., 2020 [25]	Study design: Systematic review (secondary data)
Gao et al., 2020 [26]	Data format: Aggregated data (no patient-level data available)
Solangon et al., 2022 [27]	Data format: Aggregated data
Iwahashi et al., 2017 [28]	Wrong population: Ectopic pregnancy in a non-stump site (despite previous salpingectomy)
Singhal et al., 2015 [29]	Wrong population: Ectopic pregnancy in a non-stump site (despite previous salpingectomy)
Ahmed et al., 2019 [30]	Wrong population: Cervical stump pregnancy (not tubal stump)
Nezhat et al., 2021 [31]	Wrong population: Technique description without specific stump ectopic data
Oshodi et al., 2021 [32]	Wrong population: No previous salpingectomy
Gabriel et al., 2021 [33]	Wrong population: No previous salpingectomy
Vallabh-Patel et al., 2011 [34]	Wrong population: No previous salpingectomy

Table S4 - Index data and collecting data rules

Index data	Author, Publication year, Country, Age, Gravidity, Parity, Type of fallopian tube resection, Side of fallopian tube resection, Cause of fallopian tube resection, No. of previous EP, Interval between salpingectomy and occurrence of ipsilateral (or bilateral) EP, Mode of conception, No. of transferred embryos (if IVF-ET) Type of embryo (fresh, frozen, not specified) No. of previous ET cycles GA at diagnosis, days / Time from ET heterotopic pregnancy (yes/no) if "heterotopic pregnancy: yes": type of intrauterine pregnancy (singleton/twin), Pre-treatment β-HCG, Ultrasound appearance, US Visible fetal heartbeat, US Echographic stump diameter, US Gestational sac, CRL, US Hemoperitoneum, Vaginal bleeding, Abdominal pain, Hypovolemic shock symptoms, Pregnancy side, Ruptured pregnancy, Surgical procedure, Conversion from laparoscopy to laparotomy, Surgical Treatment, Medical Treatment, Vasopressin/argipressin, Internal bleeding, Preoperative/pre-treatment Hb, Hematocrit, Blood pressure, Pulse rate, Outcome of intrauterine pregnancy, Mode of cesarean section, Gestational Age at delivery, Healthy baby, Birth weight.
Collecting data rules	When the number of days or the number of months were not specified, we considered what was indicated (e.g. 1 year = 12 months, 1 week = 7 days).<br>If explicitly indicated, the exact number of months or days was considered (e.g. 5 weeks + 3 days = 38 days).<br>If the week was marked as "1/2" was then rounded down: 3 days.<br>Cases of recurrence in the same patient were considered as separate cases.<br>Heterotopic pregnancy, intrauterine pregnancy gestational age at delivery: since some studies did not provide the exact week of the gestational age of delivery of the intrauterine pregnancy, but only indicated "term", we considered a cut off of 37 weeks, obtaining two groups (1st < 37 weeks; 2nd ≥ 37 weeks and “term”).<br>When comorbidities were not expressed (Cornual resection, Cause of fallopian tube resection, No. of previous EP, No. of previous tubal EP due to spontaneous pregnancy, No. of previous tubal EP due to IVF-ET, Other previous gynecological history, PID, Positive microbial swab, Previous pelvic surgery, Smoke), they were not reported. Conversely, as regards the symptoms (Vaginal bleeding, Abdominal pain, Hypotension/shock symptoms), if not reported, they were considered absent, therefore negative. If the type of salpingectomy (total or partial) was not specified, this was considered as "unspecified", so the cases of "unspecified" salpingectomy were subjected to separate statistical processing.<br>The treatments were divided into "conservative" and "non-conservative".<br>The "non-conservative" ones, which tend to be implemented when the patient is unstable presenting a rupture of the ectopic pregnancy, hemoperitoneum, include laparoscopic/laparotomic tubal stump excision.<br>The “conservative” ones usually implemented when the patient is stable presenting a unruptured ectopic pregnancy, without hemoperitoneum, include laparoscopic/laparotomic cornuotomy, intracardiac potassium chloride (KCl) injection, local KCl and methotrexate injecton, aspiration of ectopic pregnancy, expectant management.

Table S5. Modified JBI checklist for case report and case series (tool for systematic review) [36]

Authors \ Leading questions	Was there clear reporting of the presenting site(s)/clinic(s) demographic information?	Was the patient' s history clearly described and presented as a timeline?	Was the current clinical condition of the patient on presentation clearly described?	Was the condition measured in a standard, reliable way for all participants included in the study?	Did the case series have consecutive inclusion of participants in the case series?	Was the intervention(s) or treatment procedure(s) clearly described?	Was the post-intervention clinical condition AND outcomes or follow-up results of cases clearly described?	Were adverse events (harms) or unanticipated events identified and described?	Does the reporting of the case/cases provide a takeaway lessons?
Al-sunaidi [42]	YES	NC	YES	YES	NA	YES	YES	YES	YES
Arbab [43]	YES	YES	YES	YES	NA	YES	NO	NC	NC
Baker [44]	YES	NC	YES	YES	NA	YES	YES	YES	YES
Balafoutas [45]	YES	NC	NO	YES	NA	YES	NO	NO	NC
Banzai [46]	YES	NC	YES	YES	NA	YES	YES	NC	YES
Ben-ami [47]	YES	NC	YES	YES	NA	YES	YES	YES	YES
Bhat [48]	YES	YES	YES	YES	NA	YES	YES	NC	YES
Blazar [49]	YES	NC	YES	YES	NA	YES	YES	YES	YES
Bornstein [50]	YES	NC	YES	YES	NA	YES	YES	NC	YES
Chang [14]	YES	NC	YES	YES	NA	YES	YES	NO	YES
Chen [51]	YES	YES	YES	YES	NA	YES	NO	NO	NC
Chin [52]	YES	NC	YES	YES	NA	YES	YES	NC	YES
Van der Weiden [53]	YES	NC	NO	YES	NA	YES	YES	NO	YES
Divry [54]	YES	NC	YES	YES	NA	YES	YES	NO	YES
Dumesic [55]	YES	NC	YES	YES	NA	YES	YES	YES	YES
Felemban [56]	YES	NC	YES	YES	NA	YES	NO	YES	NC
Garavaglia [57]	YES	NC	YES	YES	NA	YES	NO	NC	NC
Ji [58]	YES	NC	YES	YES	NA	YES	YES	NO	YES
Kalampokas [59]	YES	NC	NO	YES	NA	YES	NO	NC	NC
Kasum [15]	YES	YES	YES	YES	NA	YES	YES	YES	YES
Khoo [60]	YES	NC	YES	YES	NA	YES	YES	YES	YES
Ko [7]	YES	YES	YES	YES	YES	YES	NO	NO	NC
Lower [61]	YES	NC	YES	YES	NA	YES	YES	YES	YES
Lund [62]	YES	NC	YES	YES	NA	YES	YES	YES	YES
Manea [63]	YES	NC	YES	YES	NA	YES	YES	YES	YES
Maruthini [64]	YES	NC	YES	YES	NA	YES	NO	NO	NC
Melcer [65]	YES	YES	NO	YES	NA	YES	NO	NC	NC
Okamura [66]	YES	NC	YES	YES	NA	YES	YES	YES	YES

Oral [67]	YES	NC	YES	YES	NA	YES	YES	YES	YES
Pan [68]	YES	NC	YES	YES	NA	YES	YES	NC	YES
Pavic [69]	YES	NC	YES	YES	NA	YES	NO	NO	NC
Piccioni [38]	YES	NC	YES	YES	NA	YES	NO	NC	NC
Prorocic [70]	YES	NC	YES	YES	NA	YES	YES	NC	YES
Sentilhes [71]	YES	YES	YES	YES	NA	YES	YES	YES	YES
Sharif [72]	YES	NC	YES	YES	NA	YES	YES	NO	YES
Shavit [73]	YES	YES	YES	YES	NA	YES	YES	NO	NC
Sills [74]	YES	NC	NO	YES	NA	YES	YES	NO	YES
Wang [75]	YES	NC	YES	YES	NA	YES	YES	NC	YES
Xi [76]	YES	YES	YES	YES	NA	YES	YES	NC	YES
Yip [77]	YES	NC	YES	YES	NA	YES	YES	NC	NC

	Legend
YES	Yes
NO	No
NC	Not clear
N/A	Not applicable

Figure S1. Modified JBI checklist for case report and case series (tool for systematic review) PLOT

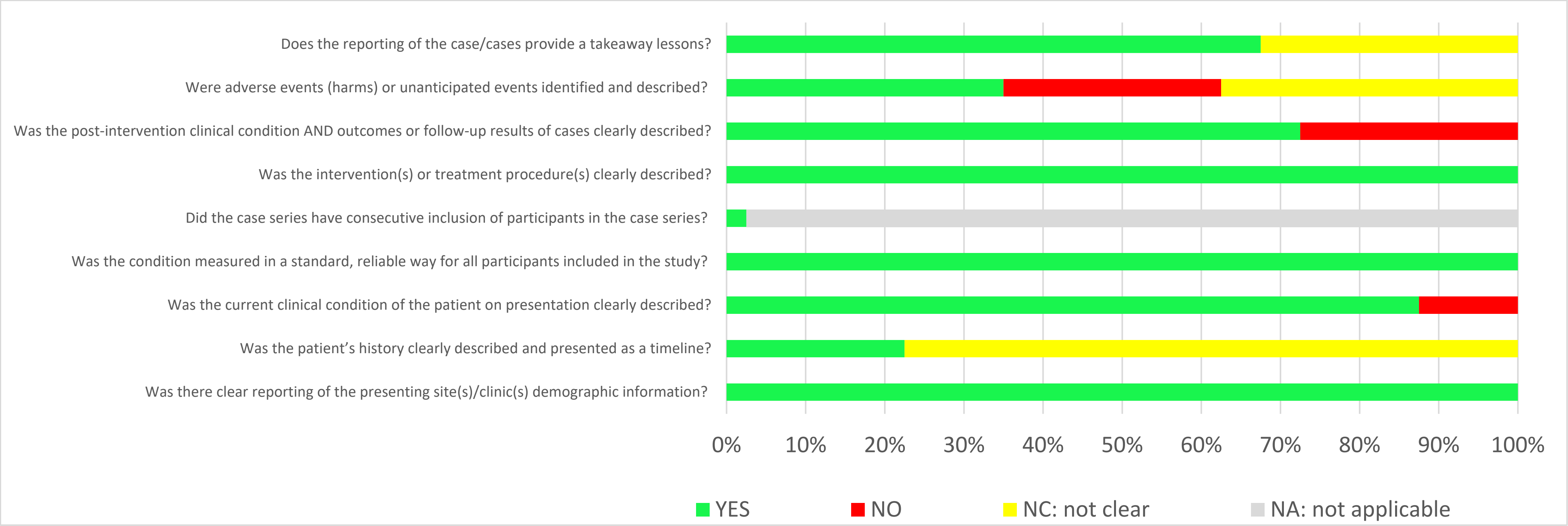

Modified from Checklist for Case Reports (2017) and Checklist for Case Series (2017) The Joanna Briggs Institute Critical Appraisal tools (Moola *et al.*, 2020) [36]
